# Comparison of Initial CT Findings and CO-RADS Stage in COVID-19 Patients with PCR, Inflammation and Coagulation Parameters in Diagnostic and Prognostic Perspectives

**DOI:** 10.5334/jbsr.2714

**Published:** 2022-07-08

**Authors:** Elif Yıldırım Ayaz, Zafer Ünsal Coşkun, Mustafa Kaplan, Ahmet Sait Bulut, Melike Yeşildal, Handan Ankaralı, Gökhan Uygun, Özge Telci Çaklılı, Mehmet Uzunlulu, Haluk Vahaboğlu, Ali Rıza Odabaş

**Affiliations:** 1 University of Health Sciences Sultan 2. Abdülhamid Training and Research Hospital, Internal Medicine Clinic, TR; 2 University of Health Sciences Sultan 2. Abdülhamid Training and Research Hospital, Department of Radiology, TR; 3 Sancaktepe Şehit Prof. Dr. İlhan Varank Training and Research Hospital, Anesthesiology and Reanimation Clinic, TR; 4 Istanbul Medeniyet University Faculty of Medicine, Department of Biostatistics, TR; 5 Göztepe Prof. Dr. Süleyman Yalçın City Hospital, Internal Medicine Clinic, TR; 6 Istanbul University, Istanbul Faculty of Medicine, Department of Internal Medicine, Endocrinology Clinic, TR; 7 Dr. Süleyman Yalçın City Hospital, Infectious Disease Clinic, TR; 8 University of Health Sciences Sultan 2. Abdülhamid Training and Research Hospital, TR

**Keywords:** COVID-19, SARS-CoV2 RT-PCR testing, chest, computerized tomography, CO-RADS, mortality

## Abstract

**Objectives:**

This study aims to determine whether COVID-19 patients with different initial reverse transcriptase-polymerase chain reaction (RT-PCR), computed tomography (CT) and laboratory findings have different clinical outcomes.

**Materials and Methods:**

In this multi-center retrospective cohort study, 895 hospitalized patients with the diagnosis of COVID-19 were included. According to the RT-PCR positivity and presence of CT findings, the patients were divided into four groups. These groups were compared in terms of mortality and need for intensive care unit (ICU). According to the COVID-19 Reporting and Data System (CO-RADS), all patients’ CT images were staged. Multivariate binary logistic regression analysis was used to examine the relationship between CO-RADS and predictive inflammation and coagulation parameters.

**Results:**

RT-PCR test positivity was 51.5%, the CT finding was 70.7%, and 49.7% of the patients were in the CO-RADS 5 stage. The need for ICU and mortality rates was higher in the group with only CT findings compared to the group with only RT-PCR positivity, (14.9% vs. 4.0%, p < 0.001; 9.3% vs. 3.3%, p > 0.05; respectively). Mortality was 3.27 times higher in patients with CO-RADS 4 compared to those with CO-RADS 1–2. Being in the CO-RADS 4 stage and LDH were discovered to be the most efficient parameters in determining mortality risk.

**Conclusion:**

Performing only the RT-PCR test in the initial evaluation of patients in SARS-CoV-2 infection may lead to overlooking groups that are more at risk for severe disease. The use of a chest CT to perform CO-RADS staging would be beneficial in terms of providing both diagnostic and prognostic information.

## Introduction

Coronavirus disease 2019 (COVID-19) has affected the entire world because of its rapid spread in the community, which causes severe acute respiratory disease, necessitating extensive medical care, high mortality rates, and the short time between onset and death [1]. The diagnosis of the disease is primarily based on the detection of the severe acute respiratory syndrome coronavirus 2 (SARS-CoV-2) virus in respiratory secretions by reverse transcriptase-polymerase chain reaction (RT-PCR) [2]. However, the negative RT-PCR test in the upper respiratory tract swab does not rule out the disease and it is important to examine the radiological images of the lung, which is the main location of the disease. There have also been reports of COVID-19 radiological findings being discovered by chance in asymptomatic patients [3]. COVID-19 patients vary in the initial clinical, laboratory, and radiological aspects. While some patients have neither computed tomograpghy (CT) findings nor RT-PCR positivity, some patients have both of them, some have only RT-PCR positivity, some have only CT findings. However, it is unknown whether these four groups of hospitalized patients have different clinical outcomes.

A wide spectrum of radiological findings has been described in COVID-19, from mild disease to severe pulmonary involvement. Hu et al. devised a chest CT severity score (CTSS) for patients based on the level of CT involvement and discovered that the greater the score, the higher the risk of mortality in patients who deteriorated [4]. Comprehensive studies that examine not just the extent of involvement, but also the relationship between disease-specific findings and mortality are needed when evaluating COVID-19 radiological findings.

The Dutch Radiology Association has developed the COVID-19 Reporting and Data System (CO-RADS) in order to categorize pulmonary involvement findings and the severity of findings according to chest CT evaluation in patients with suspected COVID-19 [5]. It is unknown whether there is a difference in the clinical outcomes of patients based on stages of the CO-RADS classification.

It is known that inflammation and coagulation markers in the initial laboratory evaluation of the patients are associated with mortality and need for the intensive care unit (ICU) [6]. The association between CO-RADS stages and patient clinical outcomes has yet to be clarified. At the time of admission to the clinic, there are patients who are at an advanced stage radiologically but whose laboratory parameters do not change negatively, as well as patients whose laboratory parameters have changed negatively but are not affected radiologically. There is a need for studies comparing the effects of CO-RADS staging and laboratory parameters on disease severity.

This study aims to determine the rates of initial RT-PCR positivity and CT findings in hospitalized patients with a diagnosis of COVID-19, to determine whether there is a difference in mortality and need for ICU between patients grouped according to the initial RT-PCR positivity and presence of CT findings, and to evaluate the effect of the CO-RADS stage on these outcomes. Moreover, it is to compare the effects of inflammation and coagulation markers and CO-RADS stage on the severity of the disease. This study hypothesizes that first, the severity of the disease is higher in patients with both CT finding and RT-PCR positivity compared to patients with either one of these, and the same is true for those with only CT findings against those with only RT-PCR positivity. Second, as the CO-RADS stage rises, so does the mortality rate and the need for ICU. Third, the CO-RADS stage is more significantly related to disease severity than predictive laboratory parameters.

## Materials and Methods

### Study Design

**Figure 1:**
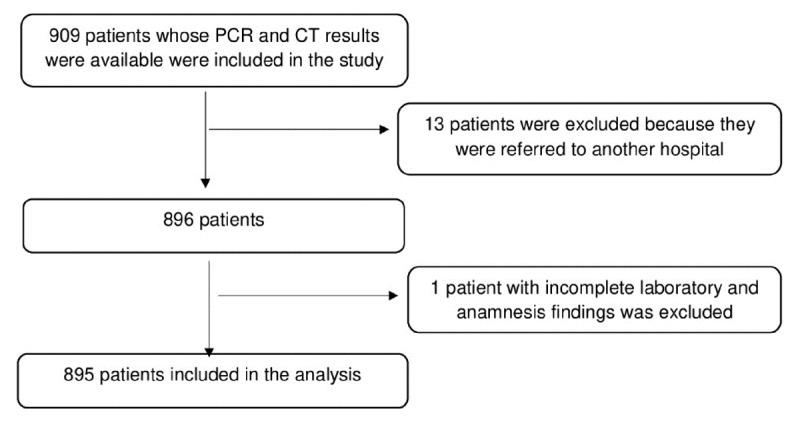
The flow diagram of patients included in the analysis.

This multi-center, retrospective cohort study was conducted at University of Health Sciences Sultan 2. Abdülhamid Training and Research Hospital and Göztepe Prof. Dr. Süleyman Yalçın City Hospital with patients who were interned with a diagnosis of COVID-19 between March 11 to June 18, 2020. Inclusion criteria were being 18 years of age or older, having SARS-CoV-2 RT-PCR test and chest CT examinations performed at the time of application or within 24 hours after admission, RT-PCR positivity in nasopharyngeal swabs sample, and/or the presence of COVID-19-compatible radiological findings in chest CT (at baseline or during the hospitalization), and being diagnosed with COVID-19. Patients without PCR positivity or CT findings at baseline and during hospitalization were excluded. Patients with insufficient or lack of image quality were not included. Patients who were transferred to another hospital were excluded because accurate outcome data would not be accessible, and patients with incomplete laboratory and anamnesis findings were also excluded. The flow diagram of the patients included in the analysis is given in Figure 1.

Before starting the study, permission was obtained from the University of Health Sciences Hamidiye Non-invasive Investigation Ethics Committee (approval number: 20/303, approval date: 09/10/2020). The patients’ consent was not required because of the retrospective nature of the study. This study was registered at the Protocol Registration and Results System (Clinicaltrials.gov PRS) with the registration number NCT04789447 (06/04/2021). This cohort study was reported in adherence to Strengthening the Reporting of Observational Studies in Epidemiology (STROBE) reporting guideline.

### Imaging Technique and Interpretation

All patients were imaged in supine position, on a 320 detector CT (Aquilion-ONE, Toshiba Medical Systems, Otawara, Japan.). The scanning protocol was the standard protocol, that is without intravenous contrast. All images were obtained in standard dose protocol with a 5 mm slice thickness in lung window setting. All images were reviewed in a standard clinical Picture Archiving Communication Systems (PACS).

Definitions of the CO-RADS stages are given in Table 1. CO-RADS 1 and 2 radiological findings were defined as negative, while CO-RADS 3, 4, and 5 radiological findings were defined as positive. Patients with CO-RADS 0, which means insufficient or lack of image quality, were already excluded from the study. CO-RADS-6 is not included in the classification of radiological images because it depends on RT-PCR positivity.

**Table 1:** COVID-19 Reporting and Data System (CO-RADS) classification.


**CO-RADS 0: Not interpretable** Poor image quality or lack of images

**CO-RADS 1: Very low** Normal CT image or non-infectious CT findings.

**CO-RADS 2: Low** Infectious manifestations specific to pathogens other than COVID-19

**CORADS-3: Equivocal/unsure** Suspicious findings in which can also be caused by other viral pneumonia or non-infectious reasons

**CO-RADS 4: High** COVID-19 findings has typical, but similar with other viral pneumonia

**CO-RADS 5: Very high** Typical COVID-19 pulmonary involvement

**CORADS-6** Proven rRT-PCR test positive


Chest CTs were subjected to CO-RADS classification in consensus by two radiologists experienced in chest radiology (reader 1: a senior radiologist with 20-year experience, reader 2: a resident radiologist 4-year experience). When there was a difference of opinion about the findings, the two researchers had a discussion and reached an agreement. Otherwise, cases were evaluated by a third reader (a senior radiologist with 13-year experience).

### Data Collection

The patients’ socio-demographic information, symptoms, comorbidities, and treatments for SARS-CoV-2 infection during their hospitalization were recorded from the hospital electronic records. The results of RT-PCR, chest CT examinations and other laboratory parameters of all patients which were performed at the time of admission were evaluated. Parameters determined as inflammation and coagulation biomarkers were recorded. The first lymphocyte, D-dimer, CRP, ferritin, fibrinogen and LDH levels within 24 hours after the first admission of the patients were evaluated.

Patients’ discharge status, whether they required ICU, and whether those admitted to ICU required invasive mechanical ventilation (IMV) were all recorded. Mortality was defined as mortality occurring during hospitalization and/or within 30 days after admission. The epicrisis of COVID-19 patients were written in detail, and all records were independently checked by two separate internal medicine specialists to verify the data. Moreover, the records of the patients until 31 December 2020 were also reviewed. Records of clinical data, interpretation of radiological findings, and statistical analysis were performed by three independent teams, all of whom were blinded to data other than the variables they examined.

### Statistical Analysis

Using the results of the initial RT-PCR positivity and presence of COVID-19 related CT findings the following groups were defined:

- PCR – CT–
- PCR + CT–
- PCR – CT+
- PCR + CT+

All radiological images are classified between CO-RADS 1–5.

Descriptive statistics of the obtained data were calculated as mean ± SD, median (25th and 75th), count, and percent frequencies. The compliance of numerical variables to the normal distribution was examined using the Shapiro-Wilks test. The association of RT-PCR and CT used in the diagnostic test with outcome variables associated with the disease, and the relationships of CO-RADS scores were analysed using Pearson’s chi-square analysis. Multivariate binary logistic regression analysis was used to assess the effects of CO-RADS and predictive laboratory parameters on mortality and the need for ICU. P < 0.05 was accepted as the statistical significance level and SPSS (ver. 23) program was used in calculations. When the studies were assessed in light of the study’s primary hypotheses, the proper sample size was determined to be 700 when the probability of making a Type I error was 5% and prior power was 80%. In the calculation, the online calculation tool on the https://www.stat.ubc.ca/~rollin/stats/ssize/caco.html website was used.

### Results

The mean age of the 895 patients hospitalized with the diagnosis of COVID-19 was 52,77 ± 20,26, 26, and 62.9% were male. Patients’ demographic characteristics, comorbidities, treatments they received for COVID-19, outcomes, and laboratory and radiological findings are given in Table 2.

**Table 2:** Demographic characteristics, comorbidities, treatments, outcomes, laboratory, and radiological findings of the participants.


		MEDIAN (IQR) OR N (%)

Age (years)		55.00 (35.00–68.00)

Lymphocyte (10^3^/μL)		1.37 (1.01–1.93)

D-dimer (ng/mL)		394.00 (199.25–1017.50)

C reactive protein (mg/L)		23.90 (6.00–75.20)

Fibrinogen (mg/dL)		487.00 (366.00–614.00)

Ferritin (ng/mL)		166.34 (76.51–403.04)

Lactate dehydrogenase (U/L)		402.00 (324.00–546.00)

Gender	Male	563 (62.9)

	Female	332 (37.1)

Diabetes Mellitus		172 (19.2)

Hypertension		283 (31.6)

Heart Failure		45 (5.0)

Cardiovascular disease		106 (11.8)

Chronic kidney disease		28 (3.1)

Chronic obstructive pulmonary disease		61 (6.8)

Asthma		39 (4.4)

Other Pulmonary Disease		11 (1.2)

Cancer		37 (4.1)

Stroke		26 (2.9)

Hydroxycloroquine		854 (95.4)

Favipravir		238 (26.6)

Azithromycin		645 (72.1)

Convalescent plasma		21 (2.39)

Lopinavir/ritonavir		63 (7.0)

Tocilizumab		24 (2.7)

Corticosteroid		50 (5.6)

SARS CoV-2 PCR	Negative	434 (48.5)

	Positive	461 (51.5)

Chest CT	Negative	262 (29.3)

	Positive	633 (70.7)

CO-RADS	1	188 (21.0)

	2	74 (8.3)

	3	87 (9.7)

	4	101 (11.3)

	5	445 (49.7)

PCR and CT	PCR:– and CT:–	112 (12.5)

	PCR:+ and CT:–	150 (16.8)

	PCR:- and CT:+	322 (36.0)

	PCR:+ and CT:+	311 (34.7)

Mortality		84 (9.4)

Need for ICU		135 (15.1)

Need for IMV in ICU		92 (68.7)


IQR: Interquartile Range, PCR: Polymerase Chain Reaction, CT: Computerized Tomography, CO-RADS: COVID-19 Reporting and Data System, ICU: Intensive care unit, IMV: Invasive mechanical ventilation.

### RT-PCR Positivity and CT Findings

**Figure 2:**
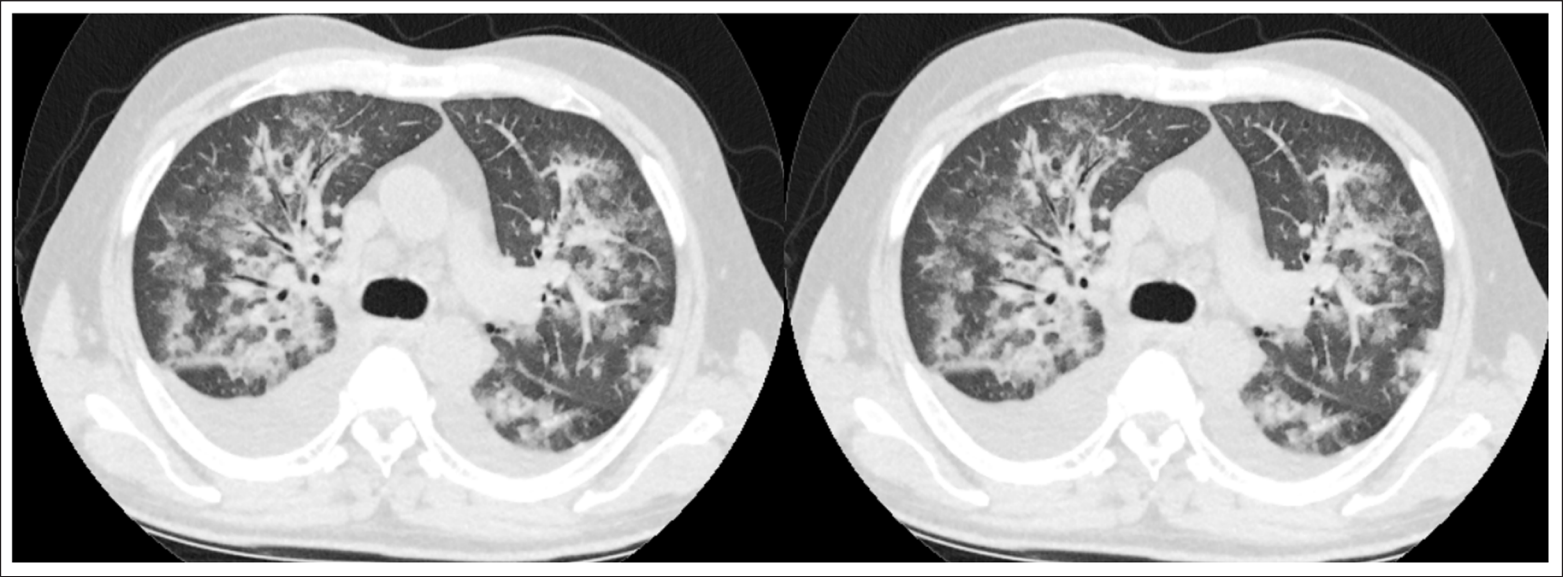
RT-PCR –, CO-RADS 5: This 56-year-old male patient had no comorbidity other than coronary artery disease. He presented to the emergency department with a fever of 38.4 degrees. He had no respiratory symptoms. There were no findings compatible with COVID-19 in laboratory parameters. RT-PCR test was negative, multifocal areas of ground glass and consolidation with bilateral pleural effusion were seen on chest CT taken in his emergency admission, and he was diagnosed with COVID-19 and hospitalized. It was evaluated as CO-RADS 5. PCR tests taken on the 1st, 5th, and 11th days of his hospitalization are also negative. The need for intensive care developed on the 11th day of his hospitalization, and he died on the 17th day.

**Figure 3:**
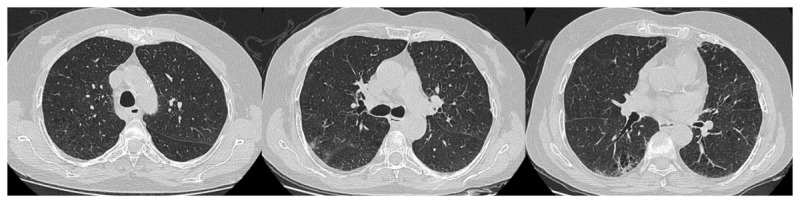
RT-PCR –, CO-RADS 4: This 70-year-old female patient had chronic obstructive pulmonary disease. He presented to the emergency department with a respiratory distress. There are no findings compatible with COVID-19 except d-dimer: 1060 ng/mL CRP: 126 mg/L in laboratory parameters. He was admitted to the intensive care unit with the diagnosis of COVID-19, with negative SARS-CoV-2 PCR test and unilateral ground glass oppacities with emphysema and bronchiectasis on chest CT. It was evaluated as CO-RADS 4. PCR tests on day 1 and day 6 are negative. He died on the 9th day of his hospitalization.

**Figure 4:**
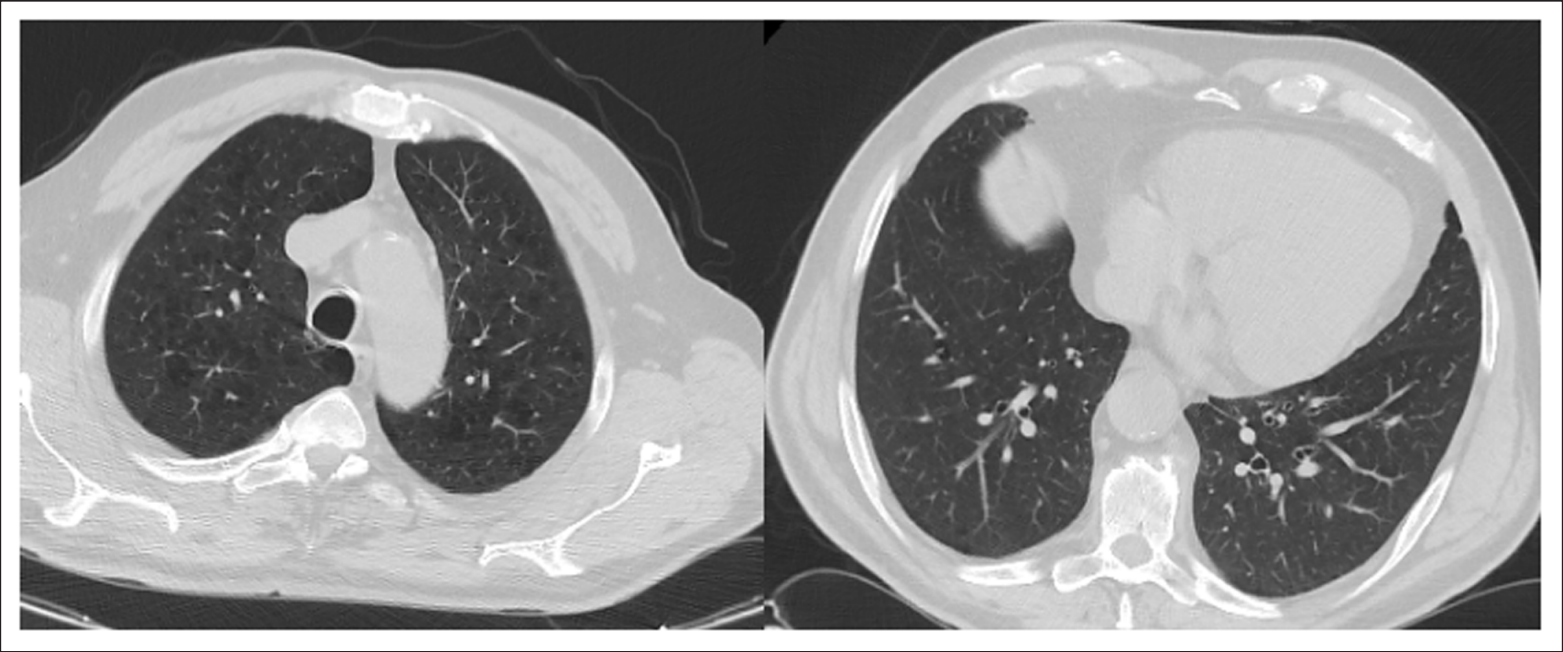
RT-PCR +, CO-RADS 1: This 65-year-old male patient had hypertension, cardiovascular disease, chronic kidney disease, and hyperlipidemia. He presented to the emergency department with shortness of breath and cough. He had hypoxemia. Laboratory parameters compatible with COVID-19 were d-dimer: 990 ng/mL, ferritin: 510 ng/mL, fibrinogen: 443 mg/dL. The patient with positive RT-PCR test and emphysematous changes in both upper lobes of the lungs on CT was hospitalized with the diagnosis of COVID-19. It was evaluated as CO-RADS 1. The patient recovered and was discharged on the 7th day.

Initial RT-PCR test positivity was 51.5%, the CT finding was 70.7%, and 49.7% of the patients were in the CO-RADS 5 stage. The radiological images of two patients with negative PCR test and positive CT findings are given in Figures 2 and 3. The radiological images of a patient with positive RT-PCR test and positive CT findings are shown in Figure 4. The clinical information of these patients is given in the figure legends.

### Comparison of Groups in Terms of Prognosis

Patients grouped according to the PCR positivity and presence of CT findings were compared in terms of mortality, need for ICU, and need for IMV among those in need of ICU (Table 3). The mortality rate was significantly higher in the PCR+ CT+ group than in the PCR+ CT- group (13.2% vs. 3.3%, p: 0.006). Mortality was higher in the group with only CT findings than in the group with only PCR positivity, but the difference was not statistically significant (9.3% vs. 3.3).

**Table 3:** Comparison of groups formed according to PCR and CT findings.


		PCR– CT–		PCR+ CT–		PCR– CT+		PCR+ CT+		P^a^
			
		N	%	N	%	N	%	N	%	

Discharge Status	Recovery	104_a,b_	92.9	145_b_	96.7	292_a,b_	90.7	270_a_	86.8	0.006*

	Exitus	8_a,b_	7.1	**5** **_b_**	**3.3**	30_a,b_	9.3	**41** **_a_**	**13.2**	

Need for ICU	No	99_a,c,d_	88.4	144_c_	96.0	274_b,d_	85.1	243_a,b_	78.1	<0.001**

	Yes	13_a,c,d_	11.6	**6_c_**	**4.0**	**48_b,d_**	**14.9**	**68_a,b_**	**21.9**	

Need for IMV ın ICU	No	3	23.1	1	16.7	14	29.8	24	35.3	0.669

	Yes	10	76.9	5	83.3	33	70.2	44	64.7	


PCR: Polymerase Chain Reaction, CT: Computerized Tomography, ICU: Intensive care unit, IMV: Invasive mechanic ventilation ^a^: Completely different letters next to the frequencies indicate columns that differ significantly. *Pearson’s Chi-Square Test* was used, * p < 0.05, ** p < 0.001.

When the groups were compared in terms of the need for ICU, it was higher in group PCR+ CT+ than in group PCR+ CT- (21.9% vs. 4.0%), and it was significantly higher in those with only CT findings than in those with only PCR positivity (14.9% vs. 4.0%) (p < 0.001).

### The Relationship of Co-Rads Staging and Laboratory Parameters with Prognosis

Mortality and ICU risk in CO-RADS 3, 4, and 5 groups were evaluated according to the sum of CO-RADS 1 and 2 groups. The combined effect of CO-RADS and laboratory parameters in determining the mortality is presented in Table 4. It was observed that being in the CO-RADS 4 stage and LDH were the most effective parameters in determining the mortality risk (those with the lowest p-value). Same parameters in determining the need for ICU is presented in Table 5.

**Table 4:** Risk factors for mortality.


	B	S.E.	WALD	DF	P	OR	95% C.I. FOR OR	

							LOWER	UPPER

CO-RADS								

3 vs (1+2)	0.129	0.404	0.102	1	0.749	1.138	0.515	2.513

4 vs (1+2)	1.185	0.392	9.127	1	0.003*	**3.270**	1.516	7.051

5 vs (1+2)	0.348	0.546	0.405	1	0.525	1.416	0.485	4.128

Lactate dehydrogenase (U/L)	0.002	0.001	9.875	1	0.002*	**1.002**	1.001	1.003

D-dimer (ng/mL)	0000	0.000	8.155	1	0.004*	**1.000**	1.000	1.000

C reactive protein (mg/L)	0.005	0.002	5.398	1	0.020*	**1.005**	1.001	1.010

Fibrinogen (mg/dL)	0.001	0.001	0.736	1	0.391	1.001	.999	1.003

Ferritin (ng/mL)	0.000	0.000	2.275	1	0.131	1.000	1.000	1.000

Lymphocyte (10^3^/μL)	-0.109	0.171	0.408	1	0.523	0.896	0.641	1.254

Constant	-4.249	0.732	33.714	1	<0.001**	0.014		


CO-RADS: COVID-19 Reporting and Data System. Multivariate logistic regression model was used. * p < 0.05, ** p < 0.001.

**Table 5:** Risk factors for intensive care unit.


	B	S.E.	WALD	DF	P	OR	95% C.I. FOR OR	

							LOWER	UPPER

CO-RADS								

3 vs (1+2)	–.288	.339	.725	1	0.394	.749	.386	1.455

4 vs (1+2)	.714	.352	4.113	1	0.043*	**2.042**	1024	4.070

5 vs (1+2)	.187	.434	.186	1	0.666	1.206	.515	2.823

Lactate dehydrogenase (U/L)	0.001	.001	5.172	1	0.023*	**1.001**	1.000	1.002

D-dimer (ng/mL)	0.000	0.000	13.418	1	<0.001**	**1.000**	1.000	1.000

C reactive protein (mg/L)	0.004	0.002	4.302	1	0.038	1.004	1.000	1.009

Fibrinogen (mg/dL)	0.001	0.001	0.494	1	0.482	1.001	0.999	1.002

Ferritin (ng/mL)	0.000	0.000	9.903	1	0.002*	**1.000**	1.000	1.001

Lymphocyte (10^3^/μL)	–0.271	0.178	2.330	1	0.127	0.763	0.538	1.080

Constant	–2.911	0.618	22.162	1	<0.001**	0.054		


CO-RADS: COVID-19 Reporting and Data System. Multivariate logistic regression model was used. * p < 0.05, ** p < 0.001.

## Discussion

In this study, in which initial CT findings and CO-RADS stage were compared with RT-PCR, inflammation, and coagulation parameters diagnostically and prognostically in hospitalized patients with the diagnosis of COVID-19, it is noteworthy that in this study, approximately half of the patients hospitalized with the diagnosis of COVID-19 had RT-PCR positivity and 70% had CT findings. The SARS-CoV-2 virus mutates like other RNA viruses and sequence variants limit diagnosis by RT-PCR [7]. Incompatibilities in primary binding regions cause decrease in test performance [8]. Moreover, virus shedding route and viral load kinetics, and improper sampling can also cause false negatives. Studies show that even best-in-class assays have a high limit of detection and false-negative rates of up to 70% in tests on the market [9]. In practice, the diagnosis of SARS-CoV-2 infection is mainly based on RT-PCR test positivity. The use of the RT-PCR test as a gold standard has been suggested in the literature as limiting pandemic control [10]. It has been shown that the sensitivity of chest CT for the diagnosis of COVID-19 is 90% and its specificity is 96% [11]. This study’s findings support the literature.

Early identification of suspected cases of COVID-19 and assessment of disease severity by CT help disease management [12]. In practice, chest CT is performed when there is clinical suspicion, although there is no specific guideline-based algorithm for this procedure [13]. Due to the radiation exposure and the burdens it places on the health system, routine use of the chest CT is not recommended unless necessary. Desmet et al. showed that low dose chest CT has a moderate to high sensitivity (75–88%) and a very high specificity (94–99%) for the diagnosis of COVID-19 [14]. Low-dose radiation CT scans will be useful in the diagnosis of the disease.

In the initial evaluation of patients, it has been proven that chest CT can detect infection-related changes within minutes, unlike PCR and serological tests, which sometimes take days to conclude, and that it is a useful tool as it provides information about prognosis and can guide treatment and follow-up decisions [15]. In the study conducted by Fonseca et al., it was found that COVID-19 patients, whose initial PCR results were negative and positive in subsequent tests, were correctly diagnosed with the CO-RADS classification according to the initial chest CT images, and thus, no patient was evaluated as false-negative [16]. In a report from a radiation oncology center, all cancer patients who received radiotherapy treatment during the COVID-19 pandemic were scanned with chest CT due to the high false-negative rates of PCR tests, and the results of the retrospectively evaluated analysis justified the necessity of scanning the patients with CT [17]. While there is no solid evidence to support CT scanning as the first line of diagnosis at this time, the growing body of evidence is adequate to encourage consideration of cases in which CT scanning should be used [18].

In this study, mortality and ICU admission rates were found to be significantly higher in the PCR+ CT+ group than in the PCR+ CT- group. While mortality and the rate of going to ICU were higher in patients with only CT findings compared to patients with only PCR positive (9.3% vs. 3.3%, 14.9% vs. 4.0%), the difference in mortality was not significant. We think that these rates were clinically significant. Based on the fact that the rates of going to ICU between the two groups were significantly different, we attribute the lack of significance of the difference in mortality to the low numbers of mortality. Severe disease rates did not change with the negative or positive RT-PCR test in patients with CT findings. Likewise, there was no difference in prognosis in patients without CT findings, depending on the RT-PCR test result. These findings show the importance of CT findings at the time of admission. While the virus is best detected in the early period of the disease with the SARS-CoV-2 RT-PCR test, the possibility of detecting the virus in secretions decreases in the later periods when the disease severity increases [19].

In this study, when compared to those in the CO-RADS 1–2 stage, patients in the CO-RADS 4 stage had three times the risk of mortality and twice the risk of being admitted to ICU. In CO-RADS 5 stage, mortality and ICU admission odds ratio (OR) values were found to be 1.4 and 1.2, respectively, compared to those with CO-RADS 1–2, but these rates were not statistically significant. An important reason for this may be the significant numerical differences in the distribution of the groups. Patients in the CO-RADS 5 stage made up half of the entire patient population. It is noteworthy that those in the CO-RADS 4 stage have a 3-fold higher mortality risk. Previous studies have found a relationship between the chest CT score and the rise in the amount of oxygen required by patients as well as the severity of the disease [20]. It is known that CTSS determined by the percentage of disease involvement in each lobe of the lung is associated with the need for ICU and mortality [21]. Van Berkel et al. showed that CO-RADS staging has higher accuracy than CTSS in the diagnosis of COVID-19 [22]. CO-RADS evaluation has been utilized as a diagnostic tool and CTSS as a prognostic tool in several studies [21]. Specificity of the radiological findings is also related to the severity of the disease. Unlike other studies, this study has shown that the CO-RADS staging system is significant in terms of disease severity.

In the current approach, laboratory parameters are frequently used to determine the prognosis of COVID-19 patients. In SARS-CoV-2 infection, prognostic models based on LDH, D-dimer, CRP, fibrinogen [23], ferritin, and lymphocyte values have been established and these models have been found to be useful in predicting the prognosis of the disease [23,24]. The lack of inclusion of chest CT results in these models is a significant limitation. In this study, the most significantly related factor with mortality was determined as LDH, followed by being in the CO-RADS 4 stage, then d-dimer and CRP. Even fibrinogen, ferritin, and lymphocyte count were not found to be associated with mortality. It is thought that the CT score directly visualizes the damage and determines the disease severity more accurately than nonspecific inflammatory markers [15]. Pulmonary involvement can be seen long before respiratory symptoms and impaired laboratory parameters. Prognostic evaluation of patients’ initial CT findings will also improve clinical outcomes [25].

This study has several limitations. First, the study has a retrospective design, which limits the extraction of causal relationships. Second, this study was conducted in two tertiary hospitals, which may have caused selection bias and limit extrapolation of results to other healthcare facilities with less severe patients. Third, mortality was less than expected, which may cause the results not to reach statistical significance for mortality, although they are significant. Fourth, when the CO-RADS staging was done, the groups showed significant numerical heterogeneity. This may limit the comparability of groups. Fifth, the study was conducted in only Turkey, this limits the generalizability of the results to other country populations.

As a result, while RT-PCR positivity was observed in only half of the COVID-19 patients admitted to the hospital, CT findings were discovered in 70%, and 70% of these patients were in the CO-RADS 5 stage, which expresses the most specific findings in terms of COVID-19. Patients with only CT findings have a poorer prognosis than patients with only RT-PCR positivity. The stage of CO-RADS is associated with mortality and the need for ICU, and this relationship is stronger than most of the predictive laboratory parameters. The most significantly related factor with mortality was determined as LDH, followed by being in the CO-RADS 4 stage. Performing only the RT-PCR test in the initial evaluation of patients in SARS-CoV-2 infection may lead to overlooking groups that are much more at risk for severe disease. CO-RADS staging is beneficial in terms of providing prognostic information as well as diagnostic information.
